# Radiotherapy in Combination With Cytokine Treatment

**DOI:** 10.3389/fonc.2019.00367

**Published:** 2019-05-22

**Authors:** Ondrej Palata, Nada Hradilova Podzimkova, Eva Nedvedova, Alexandra Umprecht, Lenka Sadilkova, Lenka Palova Jelinkova, Radek Spisek, Irena Adkins

**Affiliations:** ^1^SOTIO a.s, Prague, Czechia; ^2^Department of Immunology, 2nd Faculty of Medicine and University Hospital Motol, Charles University, Prague, Czechia

**Keywords:** radiotherapy, cytokine, immunocytokine, immunotherapy, immunogenic cell death

## Abstract

Radiotherapy (RT) plays an important role in the management of cancer patients. RT is used in more than 50% of patients during the course of their disease in a curative or palliative setting. In the past decades it became apparent that the abscopal effect induced by RT might be dependent on the activation of immune system, and that the induction of immunogenic cancer cell death and production of danger-associated molecular patterns from dying cells play a major role in the radiotherapy-mediated anti-tumor efficacy. Therefore, the combination of RT and immunotherapy is of a particular interest that is reflected in designing clinical trials to treat patients with various malignancies. The use of cytokines as immunoadjuvants in combination with RT has been explored over the last decades as one of the immunotherapeutic combinations to enhance the clinical response to anti-cancer treatment. Here we review mainly the data on the efficacy of IFN-α, IL-2, IL-2-based immunocytokines, GM-CSF, and TNF-α used in combinations with various radiotherapeutic techniques in clinical trials. Moreover, we discuss the potential of IL-15 and its analogs and IL-12 cytokines in combination with RT based on the efficacy in preclinical mouse tumor models.

## Introduction

The radiation therapy or radiotherapy (RT) started to be used as a cancer treatment modality soon after the discovery of X-rays in 1895 by Wilhelm Röntgen and Marie Curie's discovery of the radioactive elements polonium and radium in 1898. More than 100 years later, RT plays an important role in the therapy of cancer patients and represents a part of the management of more than 50% of patients during the course of their disease. RT is generally used as a primary therapy of localized tumors and regional lymph nodes in a curative setting but also as a palliative treatment to alleviate symptoms or for local control of metastasis. Ionizing radiation is frequently administered in combination with other treatment modalities such as surgery, chemotherapy, hyperthermia, hormone therapy, or immunotherapy (1, 2). RT can be administered as a neoadjuvant intervention to decrease the tumor size, intra-operatively to gain access to neoplastic lesions in a particularly complicated anatomic location or as an adjuvant treatment to prevent disease relapse (3). The most common cancer indications for RT include tumors of breast, lung, cervix uteri, endometrium, stomach, prostate, leukemia, lymphomas, skin, brain, or head and neck (4).

RT utilizes ionizing radiation that delivers its energy via photons, protons, and electrons. High doses of ionizing radiation are employed to kill tumor cells or slow their growth by inducing DNA damage and block the cell division. This process may take days and weeks of treatment before DNA is damaged enough for cancer cells to die, and the cancer cells keep dying over weeks after the termination of radiation treatment. The amount of absorbed radiation in photon RT is measured as joules per kilogram, expressed in the unit gray (Gy) and applied doses vary depending on the cancer type and stage of cancer being treated. The curative use of local ionizing radiation aims at achieving the cancer cells elimination while causing the least toxicity to normal adjacent tissues. The total dose of ionizing radiation is applied in fractions which refers to the delivery of the prescribed dose during separate radiation sessions, usually once per day (5). This provides time to normal healthy cells to recover, while tumor cells are generally less efficient in repair between fractions. Similarly, fractionation can sensitize tumor cells to RT by inducing reoxygenation or shifting the tumor cells to a radiation-sensitive phase of the cell cycle. Fractionation regimens are individualized for different clinical applications. Nevertheless, the standard fractionation schedule (“normofractionation”), which is based on extensive clinical empirical evidence, involves doses of 1.8–2 Gy per day, 5 days a week. The total cumulative dose can differ based on the tumor radiosensitivity and can range from 20 to 40 Gy for lyphomas, from 45 to 60 Gy for most tumor types to control microscopic disease after surgical resection or preoperatively in a neoadjuvant approach and from 60 to 80 Gy for curative purposes in some types of solid epithelial tumors (5). Modified fractionation schedules such as hyperfractionation or hypofractionation are also used. Hyperfractionation involves increasing the number of fractions per day while the dose per fraction becomes lower. This was shown to be beneficial in fast growing tumors such as head and neck squamous cell carcinoma (HNSCC) (6). Hypofractionation means lowering the number of fractions per weak while increasing the dose. Hypofractionation schedules are used in palliative treatments of i.e., bone metastasis (7). Similarly, radiation schedules applying single high radiation doses are employed in stereotactic radiosurgery (SRS) for brain metastasis (8).

RT comprises a wide range of various techniques which are used in dependence on the type of cancer, size of the tumor, anatomic location of the tumor, proximity of the tumor to normal tissue sensitive to radiation, or how radiation is applied to target. The use of RT techniques also depends on the patient's medical history and general health. RT can be broadly divided into 2 groups in dependence on how the radioactivity is applied to target malignant lesions: external-beam radiotherapy (EBRT) and internal radiotherapy (1). EBRT is the most common RT, which is applied on malignant lesions through the intact skin. The internal radiotherapy can be further divided into brachytherapy and systemic RT. In brachytherapy, the radiation source is placed directly at the site of the tumor. Tumors can be treated with very high doses of localized radiation with low probability of damage to the surrounding healthy tissues. This might provide an advantage over EBRT in certain clinical settings. Systemic radioisotope therapy is based on the distribution of a radionuclide or on radioisotopes attached to a tumor-targeting antibody or another tumor-targeting molecule (1). RT has several side effects which are, except for fatigue, associated with the anatomical location of irradiated volumes of the RT fields (4). RT-induced side effects can be broadly divided into early toxicities, occurring during or shortly after the end of RT treatment and late toxicities. Late toxicities occur at least 6 month after the end of RT treatment and are often irreversible (4). Over the last two decades, new RT techniques in the field have been developed and made accessible to cancer patients in routine clinical practice to improve the therapeutic efficacy of RT and to lessen the RT-related toxicities. This involves the introduction of intensity-modulated radiotherapy (IMRT), image-guided radiotherapy (IGRT), stereotactic radiotherapy (SRT), or proton or carbon beam therapy (4). These techniques can greatly increase the therapeutic efficiency by localizing the radiation effect to the target volume, steeper dose gradient, and utilization of imaging methods which leads to lower toxicity to normal tissue and shorter treatment duration (4, 9).

## Radiation-Induced Effects on Tumors and Immune System

Historically, it was thought that RT exerts immunosuppressive effects. However, in the light of recent research it has been shown that the interaction with immune system is much more complex (10). Unfortunately, the immune response against tumor cells elicited by local RT alone is mostly insufficient to eliminate all tumor cells. In successful RT treatments, beside the direct effect on the irradiated cells, there has been also observed tumor regression in sites distant to the irradiated field called abscopal effect (from the Latin *ab scopus*—away from the target) (11). The abscopal response following radiation is rare in the clinic. Despite millions of patients treated worldwide between 1969 and 2014, the abscopal effect of RT was reported only in 46 cases (11). Recently, more frequent abscopal responses were observed in patients refractory to immunotherapy with checkpoint inhibitors (ICIs) alone, who then received RT in combination with ICIs, e.g., with ipilimumab as reported by Postow et al. (12). The abscopal effect was also observed in patients undergoing RT in combination with other immunotherapeutic approaches such as cytokine therapy, Toll-like receptor (TLR) agonists or adoptive cell transfer therapy (2, 13–15).

On the molecular level, radiation causes DNA damage directly and indirectly by means of induced free radicals. Cytoplasmic double-stranded DNA (dsDNA) is detected by a cytosolic dsDNA sensor cyclic GMP-AMP synthase (cGAS). cGAS is a pattern recognition receptor that triggers IFN-I production via the downstream adaptor stimulator of interferon genes (STING) and is critical for activation of immune response to viruses (16). The RT-induced damage to the cells leads to the exposure and/or release of several damage-associated molecular pattern (DAMP) molecules such as plasma membrane-exposed calreticulin, HMGB1, and ATP during the radiation-induced immunogenic cell death (17). These molecules attract and activate dendritic cells to phagocytose dying tumor cells, to process and present released tumor antigens to T cells (17). Particularly, BATF3-tumor-infiltrating dendritic cells are stimulated by autocrine production of interferon β (IFN-β) upon detecting cell-derived dsDNA via the cGAS-STING pathway (18). Activated BATF-3-dendritic cells then migrate to tumor draining lymph nodes where they can prime CD8^+^ T cells to initiate cytotoxic T cell response. Cytotoxic CD8^+^ T cells migrate to the irradiated tumor and eliminate the residual cancer cells as well as to distant metastatic sites which can lead to a systemic tumor regression, the abscopal effect (10). Besides increasing immunogenicity of tumor cells by inducing immunogenic cell death, RT improves also the access of chemotherapeutic agents and leukocytes into the tumor sites. RT can change the immunosuppressive tumor environment by triggering expression of MHC class I, NKG2D ligands, or FAS/CD95 on tumor cells. RT can stimulate secretion of various proinflammatory cytokines or release of biologically active molecules such as reactive oxygen species and nitrogen species that can act locally to promote cell death of bystander cells (10, 19, 20). On the other hand, RT can hinder the development of anti-tumor immunity by promoting the immunosuppressive tumor microenvironment. Several mechanisms have been documented. Some cytokines, chemokines or growth factors such as tumor growth factor-β (TGF-β), the chemokine C-C motif ligand 2 (CCL2), colony-stimulating factor 1 (CSF-1), C-X-C motif chemokine ligand 12 (CXCL12), or insulin-like growth factor 1 (IRF1) are induced by RT in the tumor environment. These molecules can attract and drive differentiation of immunosuppressive populations such as M2 macrophages, myeloid-derived suppressor cells (MDSC) or directly inhibit the function of immune cells (10, 21). RT can also activate the hypoxia-inducible factor 1α (HIF-1α) which upregulates genes that control angiogenesis, metabolism and metastasis (22). Similarly, some RT regimens lead to upregulation of DNA exonuclease three-prime repair exonuclease 1 (TREX1) which cleaves RT-induced dsDNA and limits the cGAS/STING/IFN-β pathway induction (23). Interestingly, the upregulation of TREX1 was dependent on the RT regimen used. Whereas, 6 Gy and 8 Gy doses enhanced dsDNA without increasing TREX1, 20 Gy induced a prominent upregulation of TREX1 (24). This suggests that the upregulation of TREX1 is determined by the dose of a single RT fraction and not by the total dose applied. The more detailed mechanisms of RT-induced immune activation or suppression are summarized in Table 1. Radiation, specifically in combination with immunotherapy, alters the balance between immune-activating and immune-suppressive signals in the tumor microenvironment, however it seems that the success of RT is determined by the intrinsic immunogenicity of the tumor, the type of radiation dose and fractionation regimen and the type of immunotherapy agent used (10).

**Table 1:** RT-induced mechanisms that promote or limit the anti-tumor immunity.

**Immune-stimulatory effects of RT**	**Immune-suppressive effects of RT**
**Induction of DAMPs during immunogenic cancer cell death** (17) • dsDNA activates cGAS/STING pathways which leads to Interferon-β (IFN-β) production by irradiated tumor cells as well as tumor-infiltrating dendritic cells (DC), promoting the cross-presentation of antigens to T cells (18)	**Upregulation of three-prime repair exonuclease 1 (TREX1)** • leads to dsDNA degradation and impaired cGAS/STING/IFN-β pathway activation (23)
**Reoxygenation of hypoxic tumors** • an effect attributed to increased perfusion and decreased oxygen consumption (25)	**Upregulation of HIF-1α** **transcription factor** **(****25****)** • increases PD-L1 expression on tumor cells and myeloid-derived suppressor cells (MDSCs) (26) • induces VEGF-A production from tumor cells which recruits Tregs and MDSC and suppresses DC maturation (27) • increases shedding of NKG2D ligand MICA from tumor cells reducing NK cell killing (28)
**Increased secretion of chemokines and upregulation of adhesion molecules** • CXCL9, CXCL10, and CXCL16 recruit primed effector T cells to the tumor microenvironment (TME) (24, 29, 30) • adhesion molecules on tumor vascular endothelium contribute to improved T cell infiltration (31)	**Increased production of chemokines and growth factors** • CCL2 recruits monocytes to the tumor site which can be differentiated into suppressive macrophages (32) • CSF-1 enhances recruitment of MDSC (33) • CXCL12 recruits suppressive myeloid cells to TME (34)
**Upregulation of MHC class I molecules and NKG2D ligands** • in cancer cells to enable recognition by cytolytic T cells (35, 36)	**Conversion of ATP** • released from dying cells to suppressive adenosine by CD39 and CD73 expressed in TME. This leads to a suppression of DC and effector T cells while promoting Tregs and M2 macrophages (37)
**Release of tumor antigens** from dying cells • processed by DC and presented to T cells in lymph nodes (36) • presented on tumor infiltrating myeloid cells which become sensitive to cytotoxic T cell-mediated killing (38)	**Induction of senescence-associated secretory phenotype (SASP) in cancer-associated fibroblasts (CAFs)** • drives chronic inflammation and protumorogenic TME (39) • activation of insulin-like growth factor-1/receptor (IGF1/IGF-1R) promotes cancer cell growth (40) and M2 macrophage polarization (41)
**Upregulation of FAS/CD95** • to facilitate receptor-mediated apoptosis of cancer cells (42)	**Conversion of inactive TGF-β** **to an active form in TME** (43) • promotes DNA repair (44) • converts CD4^+^ T cells to Tregs, polarizes M2 macrophages and inhibits priming of CD8^+^ T cells (45)

In 2016, there were 95 clinical trials reported which examined the combinatorial effect of RT and immunotherapy. These trials included mainly ICIs, but also immunostimulatory antibodies, anti-cancer vaccines, oncolytic viruses, TLR agonists, indoleamine 2,3-dioxygenase 1 (IDO1) inhibitors, recombinant cytokines, adoptively transferred cells, and several small molecules with immunostimulatory effects (2, 46). So far, the clinical data of the effective synergy of RT and immunotherapy and reported abscopal effects are largely limited mainly to the combination of anti-CTLA-4 and RT in melanoma (2, 20, 47). Beside the ICIs, clinically significant abscopal effects were observed in a study where the combination of RT and GM-CSF in various tumor types resulted in overall response rate of 26% (11 patients out of 41) (14). The interest in cytokine therapy has been recently renewed mainly by the development of improved IL-2 analogs and IL-15 agonist being currently tested in clinical trials showing lower toxicity and improved therapeutic window (48, 49). Therefore, the main focus of this review is to summarize the data on the anti-tumor effects of combining RT and recombinant cytokines obtained in preclinical testing, but mainly in clinical trials. We discuss the effectivity of RT and cytokine treatment, and potential pitfalls and benefits together with future directions for research on RT and cytokine combinatorial treatment.

## Cytokines in Cancer Immunotherapy

Cytokines belong to a large diverse family of small glycoproteins that regulate a plethora of physiological functions in a paracrine, autocrine and endocrine manner. They play a crucial role in regulation of the innate and adaptive immunity. The development of recombinant protein technology allowed their use in modulating various pathophysiological conditions including their use in cancer treatment. Despite efforts to develop systemic anti-cancer treatment with cytokines as a standalone therapy, there are several limitations in the form of severe dose-limiting toxicities and generally low objective response rates (durable responses are approximately 10% for a systemic high dose IL-2 therapy). To circumvent this, cytokines are being investigated clinically using novel engineered cytokine mutants (superkines) or chimeric antibody-cytokine fusion proteins (immunocytokines) (50, 51).

**Figure 1:**
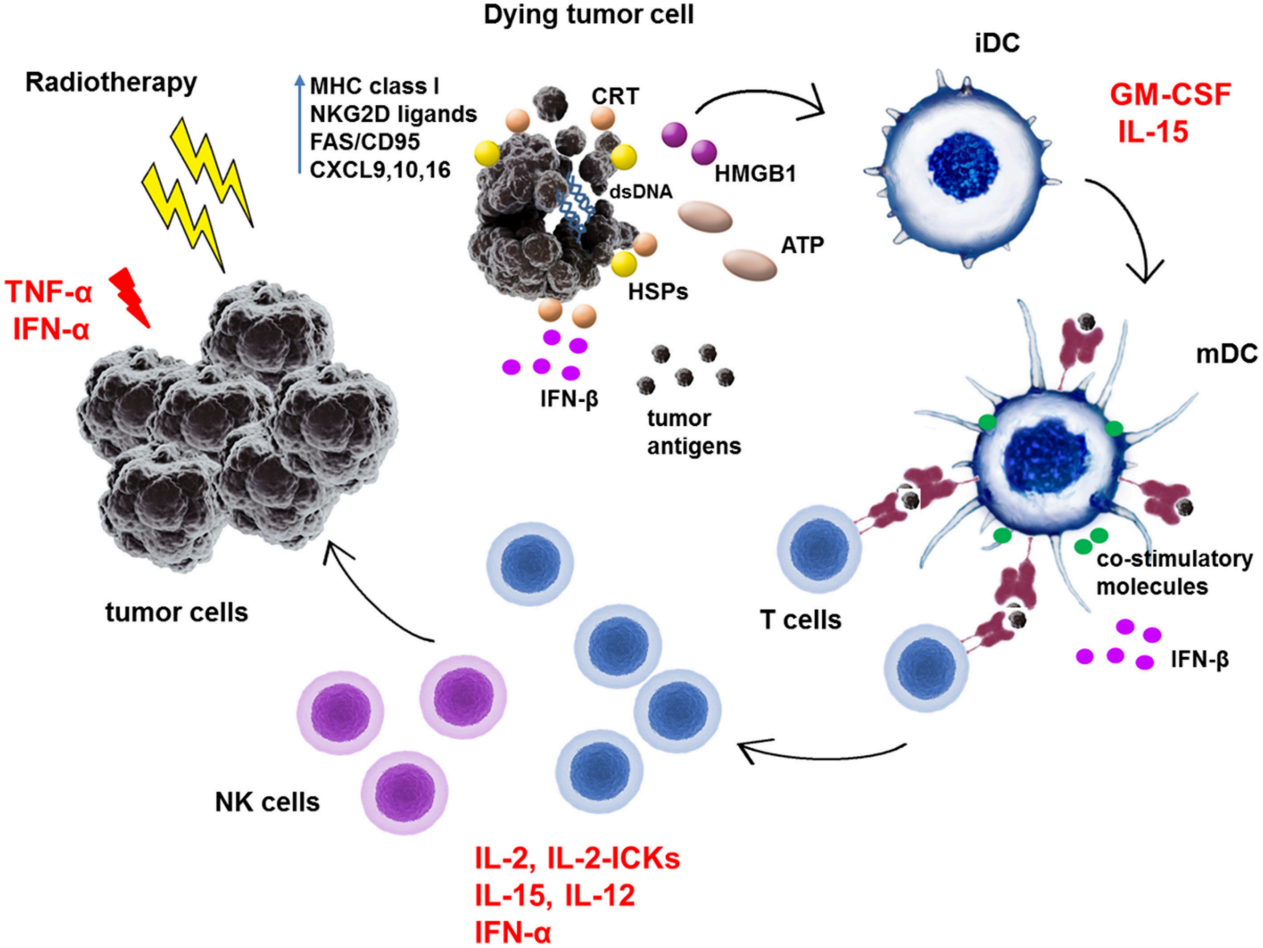
Schematic representation of immunoadjuvant effects of cytokines in combination with radiotherapy treatment of tumors. Radiotherapy was shown to stimulate anti-tumor immunity by increasing expression of MHC class I molecules, NKG2D ligands or FAS/CD95 in tumor cells. RT induces production of chemokines such as CXCL9, 10, and 16 in tumor cells which attract effector T cells to tumor site. RT induces immunogenic cell death and exposure and release of danger-associated molecular patterns (DAMPs) such as calreticulin, HMGB1, ATP, or heat-shock proteins (HSPs). Moreover, RT-generated dsDNA activates via cGAS/STING pathway the IFN-β production in tumor cells as well as in dendritic cells (DC). All of these molecules facilitate the phagocytosis of dead tumor cells and uptake of released tumor antigens by DC. They activate immature dendritic cells (iDC) to process antigens, enhance the expression of MHC class I and II molecules and co-stimulatory molecules such as CD80, CD86, CD83 on its surface to become mature dendritic cells (mDC). In lymph nodes, mDC prime T cells to become effector cells which then exert direct cytotoxic effects on tumor cells or generate the proinflammatory milieu in tumors. TNF-α and IFN-α can directly exert apoptotic effects on tumor cells. GM-CSF mainly activates dendritic cells. IL-2, IL-2 immunocytokines (IL-2-ICKs), IFN-α, and IL-15 activate directly T cells and NK cells to become cytotoxic effector cells. Moreover, IL-15 can bind to its high affinity receptor IL-15Rα on DC and as a part of immunological synapse can enhance T cell functions.

To date only few cytokines have been licensed for clinical use in a limited number of oncologic indications. Namely, recombinant IFN-α2a, IFN-α2b, interleukin (IL)-2, granulocyte colony-stimulating factor (G-SCF), granulocyte monocyte colony-stimulating factor (GM-CSF), and tumor necrosis factor α (TNF-α) (50). IFN-α2a is approved for the treatment of hairy cell leukemia and chronic myelogenous leukemia, IFN-α2b for follicular lymphoma, multiple myeloma, AIDS-related Kaposi's sarcoma, melanoma, cervical intraepithelial neoplasms, and hairy cell leukemia, and IL-2 for the treatment of metastatic melanoma and renal cell carcinoma. G-CSF and GM-CSF are approved as immunoreconstituting agents and TNF-α as an oncotoxic factor rather than to augment the anti-tumor immune responses (50). Besides the approved cytokines, there are other cytokines such as IL-12, IL-21, IL-7, IL-15, IFN-γ, IL-8, and IL-18 tested in anti-cancer treatment in clinical trials. These cytokines were tested either as monotherapy but mostly to use their immunoadjuvant potential to boost the effectivity of other therapeutic agents (50, 51). Data on the combination of RT and cytokine treatment are available only for IFN-α, IL-2, IL-15, GM-CSF, TNF-α, and IL-12, out of which the potential effects of IL-15 and IL-12 with RT have not yet been explored in patients. The summary of clinical trials combining cytokine treatment with some form of RT is shown in Table 2. The simplified immune cell-enhancing mode of action of these cytokines in combination with RT treatment is depicted in Figure 1.

**Table 2:** The summary of clinical trials combining cytokine therapy with radiotherapy.

**Agent**	**Indication**	**Phase**	**Status/Results**	**Radiotherapy**	**References**
IL-2	Metastatic melanoma	II	Completed. 2 out of 45 patients PR, 13 patients SD up to 3 months (52)	Low dose total body irradiation	ND
IL-2	Metastatic melanoma, renal cell carcinoma	I	Completed. 1 out of 12 patient CR, 7 patients PR (53)	SABR	ND
IL-2	Metastatic tumors	I	Completed. 6 out of 28 patients showed significant shrinkage of tumor (54)	Fractionated radiotherapy	ND
IL-2	Metastatic renal cell carcinoma	II	Active	SABR	NCT01896271
IL-2	Metastatic renal cell carcinoma	II	Active	SABR	NCT02306954
IL-2	Metastatic renal cell carcinoma, metastatic melanoma	II	Recruiting (55)	Booster radiotherapy	NCT01884961
IL-2	Metastatic melanoma	II	Active	SABR	NCT01416831
L19-IL2	Oligometastatic solid tumors	I	Completed. Results not published.	SABR	NCT02086721
L19-IL2	NSCLC Stage IV	II	Withdrawn (Not yet submitted, unclear timelines)	SABR	NCT02735850
NHS-IL2	Lung cancer, NSCLC	I	Completed. No objective response, 2 out of 13 patients achieved long-term survival (56)	Fractionated radiotherapy	NCT00879866
IL-2, pembrolizumab	NSCLC, metastatic melanoma, metastatic renal cell carcinoma, head and neck carcinoma	I/II	Not yet recruiting	Hypofractionated radiotherapy	NCT03474497
IL-2, ICB	Metastatic NSCLC	I	Recruiting	Hypofractionated radiotherapy	NCT03224871
IL-2, autologous DC vaccine	Renal cell carcinoma	II	Recruiting	Booster radiotherapy	NCT03226236
GM-CSF	Metastatic cancers	II	Completed. 11 out of 41 patients showed abscopal responses (14)	Not specified	NCT02474186
GM-CSF	Hepatocellular carcinoma	II	Recruiting	Carbon ion RT	NCT02946138
GM-CSF, temozolomide	Glioblastoma multiforme	II	Recruiting	Hypofractionated IMRT	NCT02663440
GM-CSF, thymosine 1 alpha	Stage IV NSCLC	II	Recruiting	SABR	NCT02976740
GM-CSF, Poly I:C	Recurrent glioblastoma	I	Not yet recruiting	Not specified	NCT03392545
Oncolytic virus expressing GM-CSF, cisplatin	Squamous cell head and neck cancer	I/II	Completed. 4 out of 17 patients CR, 10 patients PR (57)	Fractionated radiotherapy	ND
GM-CSF, vaccine therapy	Liver metastases	I	Completed. No results published.	External beam radiotherapy	NCT00081848
IL-2, GM-CSF, poxviral vaccine encoding PSA	Prostate cancer	II	Completed. 13 out of 17 patients had increases in PSA-specific T cells compared to RT alone (58)	Not specified	NCT00005916
GM-CSF, IMA950 multi peptide vaccine	Glioblastoma multiforme	I	Completed. 36 out of 40 patients had tumor antigen-specific T cells (59)	Not specified	NCT01222221
GM-CSF, pembrolizumab	Follicular lymphoma	II	Recruiting	Local radiotherapy (1 × 8 Gy)	NCT02677155
GM-CSF, pembrolizumab, GVAX	Pancreatic cancer	II	Recruiting	SBRT	NCT02648282
IFN-α, DC vaccine	Metastatic melanoma stage III–IV	II	Recruiting	IMRT-IMAT	NCT01973322
IFN-α, retinoic acid	Cervical cancer	II	Completed. No survival benefit compared to RT alone (60)	Not specified	NCT01276730
IFN-α	Melanoma	I/II	Completed. No results published	Not specified	NCT00005615
IFN-α	Melanoma	III	Completed. No results published.	Not specified	NCT00003444
IFN-α, busulfan, cellular therapy	Multiple myeloma and plasma cell neoplasm	II	Completed. No results published.	Not specified	NCT00003195
IFN-α, cisplatin,	Malignant mesothelioma	I	Completed. No results published.	Not specified	NCT00003263
IFN-α, cisplatin, 5-fluorouracil	Pancreatic cancer	II	Completed. Improved 2-year OS (61)	External-beam radiotherapy	NCT00059826
IFN-α, cisplatin, 5-fluorouracil	Esophageal cancer	I/II	Completed. 33 out of 41 patients had pathological response. Improved median survival in responders (62)	External-beam radiotherapy	ND
IFN-α, cisplatin, 5-fluorouracil	Pancreatic cancer	II	Completed. 2-year OS 84% compared to 54% in chemoradiation alone (63)	External-beam radiotherapy	ND
TNF-α	Soft tissue sarcoma	I	Completed. 2 patients out of 13 CR, 9 PR, 1 patient SD (64)	Fractionated radiotherapy	ND
TNF-α	Solid tumors	I	Completed. 5 patients out of 30 CR, 9 PR, 7 MR (65)	External beam radiation	ND
TNF-α, 5-fluorouracil, hydroxyurea	Head and neck cancer	I	Completed. 5 patients out of 12 CR, 5 PR, 2 patient SD (66)	3D conformal or IMRT	NCT00496535
TNF-α	Locally advanced, recurrent, or metastatic solid tumors	I	Completed. Only a protocol available at https://www.liebertpub.com/doi/pdf/10.1089/104303401750214320	Not specified	ND
TNF-α, 5-fluorouracil, cisplatin	Esophageal cancer	I	Completed. 6 patients out of 24 CR (67)	Fractionated radiotherapy	NCT00051480
TNF-α	Metastatic melanoma	II	Completed. No results published.	Not specified	NCT00261404
TNF-α	Rectal cancer	II	Completed. No results published.	Not specified	NCT00137878
TNF-α	Head and neck cancer	I/II	Completed. No results published.	Not specified	NCT00496236
TNF-α, 5-fluorouracil	Pancreatic cancer	III	Completed. 8 patients out of 97 PR, 72 patient SD. No survival benefit compared to Standard of Care group (68)	Fractionated radiotherapy	NCT00051467
TNF-α	Solid tumors	I	Completed. 2 out of 16 patients PR, 5 MR, 4 patients SD (69)	Not specified	ND
TNF-α, 5-fluorouracil	Pancreatic cancer	I/II	Completed. 1 patient out of 50 CR, 3 PR, 12 patients SD (70)	External-beam radiotherapy	ND

*ND, no data*.

## Interleukin 2 (IL-2)

IL-2 is a 15.5 kDa glycoprotein composed of four amphipathic α-helixes. IL-2 mediates signaling via three subunits of its IL-2 receptor which include the γ chain (γ_c_) (CD132) shared with IL-4, IL-7, IL-9, IL-15, and IL-21, the IL-2Rβ (CD122) shared with IL-15 and the IL-2Rα (CD25) chain. IL-2 signals via its high affinity receptors IL-2Rα*βγ* and via its intermediate affinity receptors, IL-2Rγβ. IL-2 promotes expansion of antigen-activated CD8^+^ T cells, acts as an important CD4^+^ T cell and NK cell growth factor and boosts antibody production in B cells. It activates NK cells and promotes differentiation and proliferation of memory CD8^+^ T cells. On the other hand, IL-2 plays a crucial role in negative regulation of T cell responses by maintaining and activating regulatory T cells and by inducing Fas-mediated activation-induced cell death (AICD) of T cells (51, 71). At low doses, IL-2 activates mainly regulatory T cells via its high affinity receptors and this might be convenient for treatment of autoimmune diseases (72). At high doses IL-2 induces cytolytic activity of T cells and NK cells involving also signaling via its intermediate affinity receptors on target cells (73). IL-2 was approved by FDA for immunotherapy of metastatic renal cell carcinoma in 1992 and metastatic melanoma in 1998 (48).

Several studies mainly in metastatic melanoma or renal cell carcinoma have been conducted over the last two decades combining IL-2 and various doses and techniques of RT, however, generally with a low efficacy or partial responses.

The combination of rapid fractionation radiation up to 20 Gy followed within 24 h by IL-2 treatment in 28 metastatic patients showed a good tolerability. Four patients showed a significant shrinkage of the tumor at the irradiated site and 2 patients showed an abscopal effect outside the irradiation field (54). Low-dose total body irradiation exhibited a synergistic immune-mediated anti-tumor effect when used in combination with IL-2 in a murine metastatic malignant melanoma model (74–76). Based on this preclinical data a phase II clinical trial combining IL-2 with RT was conducted in metastatic melanoma (52). Forty-five patients received a maximum of 2 cycles of high dose subcutaneous IL-2 and low-dose total body irradiation (single radiation fraction of 0.1 Gy on days 1, 8, 22, and 30). Of note, 0.1 Gy total body irradiation is not comparable to clinical RT. The treatment was well-tolerated but the clinical efficacy was low. In this study, an increase in percentage of cells expressing IL-2Rβ (CD122), an increase in NK cells and a decrease of B cells and monocytes was observed (52). A pilot study assessing the safety and response rate combination of SBRT followed by a high-dose IL-2 regimen in patients with metastatic melanoma and renal cell carcinoma showed complete response or partial response in 8 out of 12 patients (53). Currently, there are two ongoing phase II studies to assess the treatment with SABR in combination with a high dose IL-2 regimen for patients with metastatic renal carcinoma (NCT01896271, NCT02306954), one phase II trial for patients with metastatic melanoma (NCT01416831) and one phase II study for both patients with metastatic melanoma and renal cell carcinoma (55) (NCT01884961). Several additional clinical studies examining SABR in combination with IL-2 in metastatic melanoma and renal cell carcinoma are underway (Table 2).

Triple combinations of IL-2 administration, RT and immune checkpoint blockade is currently designed in two clinical trials as well as IL-2 and RT combined with autologous DC vaccine (NCT03226236) (Table 2). Nivolumab and Ipilimumab with IL-2 and RT will be tested in pilot phase I trial for patients with metastatic NSCLC (NCT03224871) and pembrolizumab in phase I/II trial for patients with various metastatic tumors (NCT03474497).

In recent years, new derivatives of IL-2 such as PEGylated IL-2 (NKTR-214) or IL-2 conjugated with tumor-targeting antibodies (IL-2-based immunocytokines) have found their way to the clinical testing (77, 78). More preclinical data is needed to evaluate the potential of NKTR-214 and RT combination. Currently there are no clinical trials in progress to combine NKTR-214 with RT, but some data have been already collected for IL-2 based immunocytokines.

## IL-2-based Immunocytokines

IL-2-based immunocytokines tested with RT involve L19-IL2 and NHS-IL2. Both immunocytokines showed promising results in preclinical models (79, 80). L19-IL-2 (Darleukin) is a conjugate of IL-2 and L19 an antibody fragment targeting extracellular domain B of fibronectin (ED-B). L19-IL-2 in combination with RT showed efficacy in preclinical mouse models (81–83). A long-lasting synergistic effect was observed in C51 colon tumor model with 75% of tumors cured (82). The induction of an abscopal effect was observed as well as an increase in memory CD44^+^CD127^+^ T cells. These preclinical findings set base for the initiation of phase I clinical trial in patients with metastatic solid tumors (NCT02086721), and phase II trial for stage IV NSCLC patients (currently withdrawn) (NCT02735850) (Table 2).

NHS-IL2 (selectikine) is an IL-2-based conjugate of a human antibody (NHS76) targeting necrotic tissue (non-membrane-enclosed DNA/histone complexes) fused to genetically modified human IL-2 which selectively targets the high affinity IL-2 receptor (84). Similarly to L19-IL-2, NHS-IL2 was tested in LLC lung carcinoma animal model to examine the efficacy when combined with RT and cisplatin. Mice were treated with NHS-IL2 alone (5 mg/kg; days 7–9), fractionated radiation (3.6 Gy; days 0–4) plus cisplatin (4 mg/kg; day 0), or the triple combination. Tumor regression was observed in 80% of mice when treated with RT and NHS-IL2 and in almost 100% mice when treated with the triple combination (84). Based on these results, a phase I clinical trial in patients with stage IV NSCLC (NCT00879866) was conducted. Patients received local irradiation (5 × 4 Gy) of a single pulmonary nodule. Dose-escalated NHS-IL2 was administered as 1 h intravenous infusion on three consecutive days every 3 weeks. The treatment was well-tolerated and in 2 out of 13 patients it achieved long term survival (84).

## Interleukin 15 (IL-15)

Interleukin I5 (IL-15) is a 15 kDa cytokine structurally similar to IL-2. It belongs to the four-α-helix bundle family of cytokines. The IL-15 receptor involves the γ_c_ subunit, IL-15Rβ shared with IL-2 and IL-15 specific subunit IL-15Rα. Mainly monocytes, macrophages and dendritic cells produce IL-15. This cytokine induces proliferation of various effector cells including NK cells and CD8^+^ T cells via mechanism called trans-presentation (85, 86). Soluble IL-15 binds to its IL-15Rα subunit located on the surface of antigen presenting cells, mainly dendritic cells, and then it is ligated to IL-15Rβγ receptors on target cells. IL-15 also supports the IgG production from B cells and activation and maintenance of memory CD8^+^ T cells. Even though IL-15 displays a similar effect on immune cells as IL-2, there are major differences. Unlike IL-2, IL-15 exerts anti-apoptotic effects on cells and does not expand regulatory T cells (51). IL-15 is the only cytokine found to correlate with the progression-free survival in colorectal cancer patients and with immune cell density within tumors (87). The NCI review listed IL-15 as the most promising cytokine among 12 other immunotherapeutic agents that could potentially cure cancer (88).

Recombinant human IL-15 has been tested in clinical trials as a monotherapy (89, 90), but no patient data are available on its combination with RT. In preclinical research it has been shown that IL-15 can potentiate immune activation induced by RT (91). Poorly immunogenic TSA breast cancer tumors were treated with RT (locally in 8 Gy fractions on days 13, 14, and 15), IL-15 (2 μg/mouse daily for 10 days starting on day 12), or a combination of RT and IL-15. The highest survival was observed in the RT and IL-15 combination group (median 102 days) with 1 of 6 mice showing complete tumor rejection and a development of a long-lasting immunity. Moreover, a significant infiltration of T cells was detected (91).

Similarly to IL-2, there have been various analogs of IL-15 developed to increase the anti-tumor efficacy and lower the toxicity (49). ALT-803 is a mutated IL-15 (N72D) to enhance its biological activity bound to an IL-15RαSu/Fc fusion protein (92, 93). ALT-803 has been evaluated for safety and efficacy in a few clinical trials (94, 95) including combinatorial clinical trials with ICIs. However, the data on the combination of ALT-803 and RT are available only from one preclinical study (96). Here ALT-803 was combined with a SRS in murine glioblastoma model. However, no synergistic effect was observed. More data is necessary to evaluate the effectivity of IL-15 or IL-15 analogs in cancer treatment in combination with RT.

## Granulocyte Macrophage Colony-Stimulating Factor (GM-CSF)

Granulocyte macrophage colony-stimulating factor (GM-CSF) is a 23 kDa glycoprotein that binds to a heterodimeric receptor which consists of subunits belonging to the type 1 cytokine receptor family (51). GM-CSF stimulates production of monocytes, neutrophils, and eosinophils. It is produced by various types of cells including T and B lymphocytes, neutrophils, eosinophils, epithelial cells, fibroblasts, and other cells (97). GM-CSF stimulates antigen presentation to the immune system by directly acting on dendritic cells and macrophages (98). GM-CSF was also shown to stimulate the capacity of neutrophils, macrophages and monocytes to mediate antibody-dependent cytotoxicity (51). In contrast to data from preclinical mouse models, the adjuvant effects of GM-CSF in human trials were inconsistent. This may be explained by the capacity of GM-CSF on one hand to stimulate dendritic cells, and on the other hand also to induce myeloid suppressor cells (99).

However, preclinical data show that GM-CSF in combination with RT can help boost the abscopal effect (100). Similarly, the abscopal effect of GM-CSF and RT in a patient with metastatic pancreatic cancer has been documented (101). Based on the preclinical data there had been several clinical trials conducted investigating combination of RT and GM-CSF. A proof-of-principle trial showed that 11 out of 41 patients with various metastatic diseases, developed abscopal responses (14) (NCT02474186). This trial set base for an ongoing phase II trial combining carbon ion RT and GM-CSF for patients with hepatocellular carcinoma (NCT02946138). GM-CSF in combination with RT is also combined or planned to be combined with additional agents (Table 2). GM-CSF is planned to be combined with pembrolizumab and RT (1 × 8 Gy) in follicular lymphoma (NCT02677155) or with pembrolizumab, GVAX (GM-CSF gene-transduced tumor cell vaccine) and SBRT in patients with locally advanced pancreatic cancer (NCT02648282). There is a phase II study investigating combination of GM-CSF with hypofractionated IMRT and temozolomide for patients with glioblastoma multiforme (NCT02663440). Another immune enhancer, thymosine-α, is investigated with this combination of GM-CSF and SABR in phase II trial for patients with stage IV NSCLC (NCT02976740). Similarly, in glioblastoma there is a study planning the intratumoral addition of polyI:C together with GM-CSF and RT (NCT03392545). A phase I/II study combining chemoradiotherapy with a herpes simplex type 1 oncolytic virus expressing GM-CSF in patients with HNSCC was conducted (57). The treatment was well-tolerated, 14 out of 17 patients showed response to the treatment and pathologic complete remission was confirmed in 93% of patients at neck dissection. GM-CSF treatment was also used in combination with RT and PSA tumor antigen-encoding poxviral vaccines (58) or with multipeptide vaccines (59) where an increase of antigen-specific T cells was detected in comparison to control arms.

## Interferon Alpha (IFN-α)

Interferon alpha (IFN-α) is member of the type I interferon family and acts as immune modulator with antiviral and anti-proliferative properties (102, 103). Twenty IFNs have been identified in humans, out of which the most subtypes belong to the IFN-α group. Type I IFNs signal via a common pair of receptors, IFNAR1 and IFNAR2. IFN-α is produced mainly by plasmacytoid dendritic cells but also by most of other cell types in response to encounter with DAMPs by pattern recognition receptors (PRRs) which can be produced by virus-infected or cancer cells (104, 105). IFN-α induces MHC class I expression on tumor cells, induces apoptosis of tumor cells, mediates maturation of dendritic cells and activation of B and T cells, and displays antiangiogenic properties (51).

IFN-α has been approved by FDA for treatment of several malignancies including hairy cell leukemia, chronic myelogenous leukemia, follicular lymphomas, malignant melanoma, multiple myeloma, or renal cell carcinoma (106, 107) but displays also significant toxicity and side effects such as flu-like symptoms, anorexia, fatigue, depression. It has been shown in *in vitro* models that IFN-α has a synergistic cytotoxic effect with chemotherapy and RT (108, 109). This synergistic effect as well as radiosensitizing effect of 5-fluorouracil was also observed in patients with small cell lung cancer and anal cancer (110, 111). A phase I/II study combining 5-fluorouracil, cisplatin, IFN-α and concurrent EBRT before resection in patients with advanced esophageal cancer resulted in 80% of the patients responding to the therapy but the authors claimed that the contribution of IFN-α to the treatment was uncertain (62). In a preliminary phase II study, patients with pancreatic cancer underwent similar adjuvant therapy of 5-fluorouracil, cisplatin, IFN-α and RT after pancreaticoduodenectomy (63). The study showed better survival in the group of patients receiving IFN-α in comparison with patients with similar adjuvant therapy without IFN-α. In a similar multicenter phase II study (NCT00059826), patients with pancreatic cancer undergoing this adjuvant therapy had better overall survival results but the study had to be terminated prematurely due to the high toxicity of treatment (61). Acceptable toxicity was observed in phase II clinical trial (NCT01276730) where patients with stage III cervical cancer were treated with RT in combination with IFN-α and retinoic acid (60). Unfortunately, there was no survival advantage in comparison with the group receiving RT and cisplatin. In melanoma, there have been several trials examining treatment with IFN-α and various forms of RT with mixed outcomes involving high toxicity of the combinatorial treatment as well as generally low efficacy. These studies are summarized extensively in the review of Barker and Postow (112).

A phase II study investigating the combination of RT and IFN-α with a dendritic cell-based vaccine for patients with metastatic melanoma is currently ongoing (NCT01973322). Two other clinical studies combining chemotherapy and/or cellular therapy and IFN-α have been completed (NCT 00003195 and NCT00003263) (Table 2).

## Tumor Necrosis Factor α (TNF-α)

Tumor necrosis factor alpha (TNF-α) is a strong proinflammatory cytokine with anti-tumor activity both *in vitro* and *in vivo* (113, 114). TNF-α is produced mainly by macrophages, granulocytes and epithelial cells but also by other types of cells and its anti-tumor properties are due to direct cytotoxic and antiangiogenic effects (70). Also it has been shown that TNF-α acts as a radiosentitizer and enhances cytotoxic effect of radiation (115). Despite of its anti-tumor properties, the systemic administration of TNF-α in a sufficient dose is associated with a high toxicity (116, 117). Thereby the use of TNF-α in cancer therapy is limited to isolated limb perfusion (ILP) of advanced melanoma and soft tissue sarcoma (118, 119). The only clinical testing of TNF-α and RT involves gene therapy delivering TNF-α gene to cancer cells—TNFerade™ (120, 121). TNFerade is an adenovector containing TNF-α gene with early growth response gene (Egr-1) radiation activated promoter that is injected intratumorally. In preclinical models this combination showed remarkable anti-tumor effects with minimal toxicity (122). Several trials combining TNFerade and RT or chemoradiotherapy were conducted in patients with various types of tumors including breast, lung, pancreatic, head and neck, rectal cancer, melanoma, esophageal cancer, or soft tissue sarcoma (64–67, 69, 70, 123, 124). These studies showed that the treatment is well tolerated with complete or partial tumor responses and complete tumor regressions in some patients (Table 2). Despite these favorable results a phase III clinical trial randomizing patients with locally advanced pancreatic cancer to 2:1 groups standard of care (SOC) plus TNFerade vs. SOC alone showed no survival benefit of the patients in SOC plus TNFerade group (68). This dampened the enthusiasm of using this approach and there are currently no open clinical trials (Table 2).

## Interleukin 12 (IL-12)

IL-12 is a four-bundle α-helix heterodimeric cytokine encoded by two genes: *IL-12A* (p35 subunit) and *IL-12B* (p40 subunit). The active IL-12 forms a heterodimer of p35 and p40 subunits referred as p70. The receptor for IL-12 consists of IL-12Rβ1 and IL-12Rβ2. IL-12 is produced by macrophages, dendritic cells and B cells. IL-12 induces proliferation of T cells and NK cells as well as their IFN-γ production. IL-12 polarizes Th1 immune response and displays antiangiogenic properties (51). The anti-tumor efficacy of IL-12 was shown in several animal models (125–127). IL-12 has shown promising results in preclinical studies but the clinical trials did not result in satisfactory outcome. As IL-12 displays a high systemic toxicity, the local treatment in the form of gene or viral therapy was tested in combination with RT in preclinical models (128, 129). A non-viral murine IL-2 and IL-12 gene therapy and external beam radiation (2 × 1 Gy) was tested in HNSCC in an orthotopic murine model (130). A significant increase in anti-tumor effects and T lymphocyte infiltration was detected in comparison to single therapies and the control. Furthermore, the anti-tumor and anti-metastatic activity of the oncolytic adenovirus expressing IL-12 and GM-CSF injected intratumorally in combination with RT was investigated in a murine hepatic cancer (HCa-I) model (131). This combinatorial therapy was effective in suppressing primary tumor growth and an increased immune cell infiltration was observed. The therapeutic effect of the naked IL-12 cytokine combined with fractionated RT was investigated in Lewis lung carcinoma mouse model (132). The treatment was effective against primary tumor and the number of lung metastasis decreased. A pronounced tumor growth delay was observed when GM-CSF was added together with IL-12 and fractionated RT. IL-12, similarly to IL-2, has been fused to an antibody tumor-necrosis targeting IgG1 (NHS76) to create a novel immunocytokine NHS-IL12 (133). NHS-IL12 immunocytokine exhibited a longer half-life and a selective tumor targeting *in vivo*. NHS-IL12 showed a superior anti-tumor effect when combined with RT in MC38 mouse colorectal cancer model (133). Currently there are no clinical trials combining IL-12 cytokine therapy or NHS-IL12 immunocytokine with RT.

## Conclusions and Future Perspectives

Cancer immunology has made a remarkable progress, which led to the development of various immunotherapies that can be combined with ionizing radiation. The combination of RT and immunotherapy represents a growing field of clinical investigation with an increasing number and various types of clinical trials (2). Despite partial therapeutic success of the combination of RT and immunotherapy including the rare abscopal effect, most patients do not respond to RT and immunotherapy, and the same goes for using cytokine adjuvant treatment and RT. For RT itself, there are important challenges to overcome and there is a need to conduct rigorous research. These include the selection of appropriate radiation dose, fractionation, appropriate technique for RT, sequencing of therapies and selection of meaningful endpoints in clinical trials (134). Radiation dose and regimen is likely to be a critical determinant in successful generation of an anti-tumor response. Radiation dose and regimen largely affect both the immunomodulatory and cytotoxic effects of RT. These might attenuate the immunosuppressive environment but might not induce the immunogenic cell death of cancer cells to elicit strong anti-tumor responses. In the same line, although the cytokine therapy as documented with IL-2 can induce significant durable responses in patients, there are strong limitations, which lie mainly in the toxicity after the systemic administration. This can be mitigated by intratumoral administration of cytokines, which on the other hand, might represent technical challenges for the clinicians, or targeted versions of cytokines (e.g., immunocytokines). Out of the clinically tested cytokines IL-2, IFN-α, GM-CSF, and TNF-α, overall only IL-2 and GM-CSF combinatorial treatment with RT showed some even clinically relevant efficacy with acceptable toxicity. Novel analogs of IL-2 engineered to mitigate the toxic effects and to reduce the induction of immunosuppressive T regulatory cells might increase the efficacy of treatment with radiation. Similarly, analogs of IL-15 showing promising results in clinical trials when combined with ICIs hold the potential to boost the immunogenic effect of RT. From this summary, it becomes clear that multiple combinations using cytokines and RT together with some other immunotherapeutic approaches might hold the promise to increase the clinical benefit of cancer patients. As we are only beginning to explore the possibilities of multiple immunotherapeutic combinations, the understanding how to best integrate the scientific rationale, mode of actions and the most effective therapeutic regimens remains of urgent need.

## Author Contributions

All authors listed have made a substantial, direct and intellectual contribution to the work, and approved it for publication.

### Conflict of Interest Statement

The authors are employees of Sotio a.s. None of the authors has any potential financial conflict of interest related to this manuscript.
